# Antibacterial, Antifungal and Cytotoxic Activities of Two Flavonoids from *Retama raetam* Flowers

**DOI:** 10.3390/molecules17067284

**Published:** 2012-06-13

**Authors:** Hayet Edziri, Maha Mastouri, Mohamed Ali Mahjoub, Zine Mighri, Aouni Mahjoub, Luc Verschaeve

**Affiliations:** 1 Laboratoire des Maladies Transmissibles et des Substances Biologiquement Actives, Faculté de Pharmacie, Monastir 5000, Tunisia; Email: Jaziri_hayet@yahoo.fr (H.E.); mahjoubaouni@yahoo.fr (A.M.); 2 Laboratoire de Microbiologie C H U Fattouma BOURGUIBA, Monastir 5000, Tunisia; Email: mastourimaha@yahoo.fr (M.M.); zinemighri@yahoo.fr (Z.M.); 3 Laboratoire de Chimie des Substances Naturelles et de Synthèse Organique 99/UR/12-26, Faculté des Sciences de Monastir, Monastir 5000, Tunisia; Email: medali1112@yahoo.fr; 4 Division of Toxicology, Scientific Institute of Public Health, Brussels B-1050, Belgium; 5 Department of Biomedical Sciences, University of Antwerp, Wilrijk 2610, Belgium

**Keywords:** *Retama raetam* flowers, antibacterial activity, antifungal activity, cytotoxic property, flavonoid

## Abstract

We have investigated the antibacterial, antifungal and cytotoxic activities of two flavonoids isolated from *Retama raetam* flowers using the disc diffusion and micro-dilution broth methods. The cytotoxic activity was tested against Hep-2 cells using the MTT assay. The compounds licoflavone C (**1**) and derrone (**2**) were active against *Pseudomonas aeruginosa* and *Escherichia coli* (7.81–15.62 µg/mL) and showed important antifungal activity. Strong antifungal activity against *Candida* species (7.81 µg/mL) was for example found with compound **2**. The tested compounds also showed strong cytotoxicity against Hep-2 cells. These two compounds may be interesting antimicrobial agents to be used against infectious diseases caused by many pathogens.

## 1. Introduction

The use of plant extracts in medicine, for example against microbial infections [1,2,3,4], is still very widespread and based on knowledge from traditional medicinal practice [5]. Flavonoids seem to be in very important this respect as they were found to have anti-inflammatory, antiproliferative, antiviral, antithrombotic, antimutagenic, anticarcinogenic, hepatoprotective, oestrogenic, antibacterial and antioxidant activities [6,7,8,9,10]. Antibacterial activity is widely found in chemically different groups of flavonoids. Morusin, kuwanon C, sanggenon B and D, which were isolated from *Morus* root bark showed for example strong antimicrobial activity against many microorganisms [8].

*Retama raetam* Forssk. Webb (Fabaceae) is common in the North African and East Mediterranean region and in the Sinai Peninsula [11,12]. The plant flowers from April to May. *Retama* species have been reported to contain alkaloids [13]. Flavonoids such as daidzein, vicenin-2, naringenin, apigenin, kaempferol, quercetin and kaempferol-7-*O*-glucoside are present in the seeds [14], and daidzein 7,4'-dimethyl ether, chrysoeriol 7-*O*-glucoside and orientin in the leaves [15]. Kassem *et al*. [16] have isolated two new flavonoids from the aerial part, namely luteolin 4'-*O*-neohesperidoside and 5,4'-dihydroxy-(3'',4''dihydroxy)-2",2"-dimethylpyrano-(5", 6": 7,8)-flavone. The present paper deals with the isolation of two flavonoids as well as with their antibacterial and antifungal activities and cytotoxic properties.

## 2.Results

**Figure 1:**
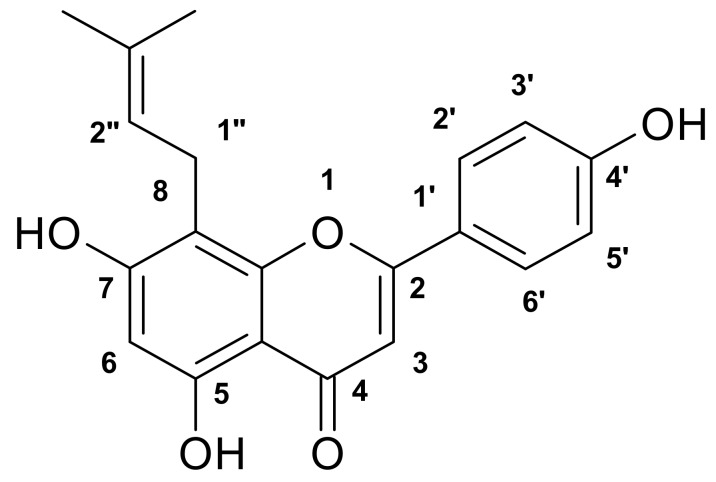
Licoflavone C (**1**).

**Figure 2:**
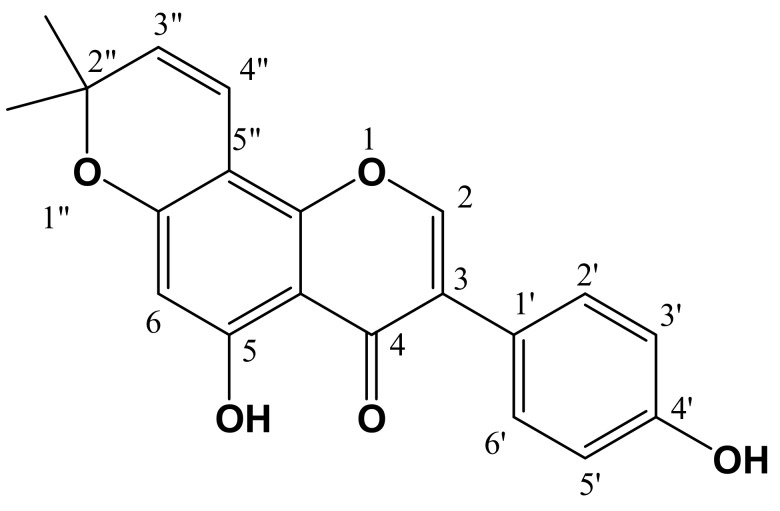
Derrone (**2**).

Column chromatography of the ethyl acetate extract of the flower of *R. raetam* yielded compounds **1** and **2**. For compound **1** (yellow powder) the molecular formula was deduced by HR-EIMS showing a molecular ion peak at 338.1154 corresponding to the molecular formula C_20_H_18_O_5_ (Figure 1). Compound **2** (brown powder) showed [M^+^] ion at 336.0998 corresponding to the molecular formula C_20_H_16_O_5_ (Figure 2). The structures were further elucidated based on the corresponding ^1^H- and ^13^C-NMR data. Compound **1** is a flavone and compound **2** is an isoflavone isolated for the first time from the flowers of *R. raetam*, although, they were previously isolated from other plants. Compound **1** was isolated from *Artocarpus communis* [17] and compound **2** was isolated from *Derris robusta* seeds [18].

Both compounds were assayed *in vitro* against *Escherichia coli* ATCC 25922, *Pseudomonas aeruginosa* ATCC 27950, *Enterococcus faecalis* ATCC 29212 and *Streptococcus aureus* ATCC 25923 for determination of their antibacterial activity. *Candida albicans* ATCC 90028, *Candida glabrata* ATCC 90030, *Candida parapsilosis* ATCC 22019 and *Candida kruseii* ATCC 6258 were used for the determination of antifungal activity. Ampicillin and ofloxacin were used as reference drugs for determination of antimicrobial activities. Antibacterial and antifungal activities were compared with the activities of the standard drugs gentamycin and amphotericin. Both compounds were found to be active in a dose dependent way Table 1. They manifested an important antibacterial activity against *S. aureus*, *P. aeruginosa* and *E. faecalis* (50 µg/disc). Compound **1** manifested the best antibacterial activity against *Escherichia coli* with an inhibition zone of 22 mm. It inhibits the growth of *E. faecalis* and *P. aeruginosa* with an inhibition zone of 20 mm. Compound **1** and **2** also showed an important antifungal activity against *Candida* species. This was especially so for compound **2** where the antifungal activity was even better than that of the positive control (inhibition zone of 25 mm).

**Table 1:** Zone of inhibition of isolated compounds of*Retama raetam*flowers.

Microorganisms	AmphotericinB ^a^	GM ^b^	Compound (1)		Compound (2)	
			25 ^c^	50 ^d^	25	50
**Bacteria**						
*Staphylococcus aureus *ATCC 25923	nd	18.3 ± 0.61	11 ± 1.10	13 ± 1.23	10 ± 2.03	12 ± 1.27
*Escherichia coli *ATCC 25922	nd	23 ± 0.02	11 ± 1.51	22 ± 1.51	17 ± 2.12	19 ± 0.23
*Enterococcus faecalis *ATTC 29212	nd	nd	16 ± 2.33	20 ± 0.98	13 ± 1.51	17 ± 0.15
*Pseudomonas aeruginosa *ATCC	nd	18 ± 0.11	14 ± 1.01	20 ± 1.8	12 ± 2.17	16 ± 0.52
**Yeasts**						
*Candida glabrata *ATCC 90030	20 ± 1.04	nd	12 ± 0.03	16 ± 1.53	20 ± 0.52	25 ± 1.11
*Candida albicans *ATCC 90028	19 ± 0.25	nd	14 ± 0.61	19 ± 0.18	21 ± 1.25	25 ± 1.24
*Candida parapsilosis *ATCC 22019	19 ± 0.51	nd	14 ± 1.72	19 ± 0.39	20 ± 1.87	25 ± 0.91
*Candida kreusei *ATCC 6258	19 ± 0.12	nd	15 ± 1.92	19 ± 0.54	21 ± 1.79	25 ± 1.63

All the values are mean values ± standard deviation of three determinations; ^a^ Amphotericin B (100 µg); ^b^ GM: Gentamycin (10 unit); ^c^ 25 µg/disc; ^d^ 50 µg/disc.

The MIC results showed that the tested compounds have lower antibacterial activity against *E. foecalis* and *S. aureus* compared with standard antibiotics (Table 2). On the other hand, compound **1** and **2** showed a good antibacterial activity against *E. coli* (MIC = 7.81 μg/mL) and moderate antibacterial activity against *P. aeruginosa* (MIC = 15.62 μg/mL).

**Table 2:** MIC of isolated compounds of *Retama raetam* flowers.

Microorganism	MIC (µg/mL)				
	CP1 ^a^	CP2 ^b^	GM ^c^	OFX ^d^	AP ^e^
**Bacteria**					
* Staphilococcus aureus* ATCC 25923	62.5	62.5	nd	0.25	nd
* Escherichia coli* ATCC 25922	7.81	7.81	nd	0.12	nd
* Enterococcus faecalis* ATCC 29212	100	100	nd	1	nd
* Pseudomonas aeruginosa* ATCC 27950	15.62	15.62	0.5	1	nd
**Yeast**					
*Candida glabrata* ATCC 90030	15.62	7.81	nd	nd	0.5
*Candida albicans* ATCC 90028	15.62	7.81	nd	nd	0.5
*Candida parapsilosis* ATCC 22019	15.62	7.81	nd	nd	0.5
*Candada kreusei* ATCC 6258	15.62	7.81	nd	nd	0.5

^a^ Compound **1**; ^b^ Compound **2**; ^c^ Gentamycin; ^d^ OFX: ofloxacin; ^e^ Amphotericin B; nd: not determined.

**Figure 3:**
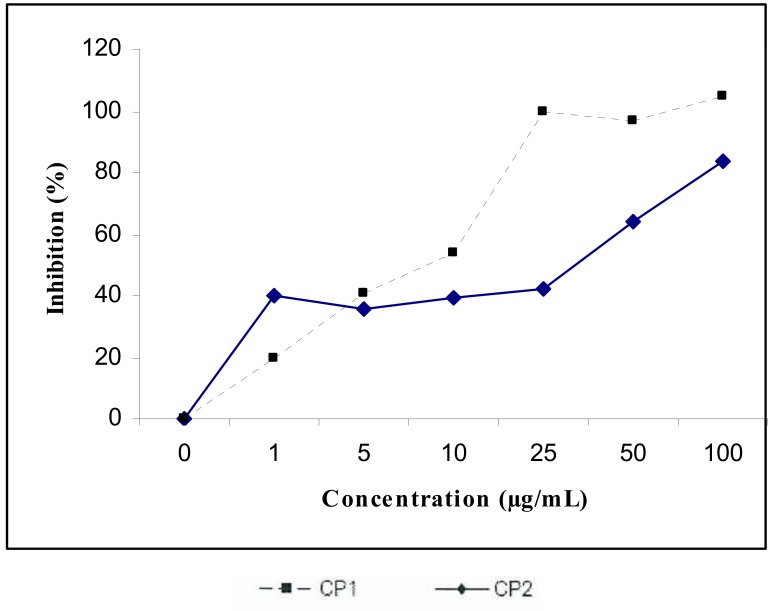
Cytotoxicactivity of compound **1** (CP1) and compound **2** (CP2) onHep-2 cells.

Cytotoxic activities according to the MTT assay are given in Figure 3. Compound **1** showed cytotoxicity against Hep-2 cells with IC50 values of 9 μg/mL, whereas this was approximately 30 µg/mL for compound **2**.

## 3. Discussion

Antibiotic resistance is a natural phenomenon to which societal factors also contribute. These factors include increased transmission of infections coupled with inappropriate antibiotic use. The use of an antimicrobial against infections, real or feared, in any dose and over any time period, forces microbes to either adapt or die in a phenomenon known as “selective pressure”. The microbes which adapt and survive carry genes for resistance, which can be passed on.

In the past few decades, more microbes have became resistant to commonly used antibiotics. These microbes are responsible for an increased number of infections and thus expand both the need for antimicrobials and the opportunities for their misuse.

There are no treatments available for infections caused by many of the antibiotic-resistant bacteria. Multiple drug-resistant organisms used in this study are common causes of infections in long-term care units in hospitals. *P. aeruginosa* is the main strain responsible for 16% of nosocomial pneumonia cases. *S. aureus* are the most important bacteria that cause disease in humans. They are the leading cause of skin and soft tissue infection [19].

The observed antibacterial activity of the two compounds against *P. aeruginosa* is novel and important. The anti-*Pseudomonas aeruginosa* activity is particularly interesting due to its importance as a nocosomial infectious agent. It developed mechanisms of resistance to common antibiotics [20].

The antimicrobial properties of phenolic compounds are well known [21,22]. Cowan showed that flavonoids serve as plant defence mechanism against pathogenic microorganisms [23]. In fact, the site and the number of hydroxyl groups determine the toxicity against the microorganisms. Tsuchia *et al*. [24] linked the antimicrobial effects of flavonoids to their capacity to form complexes with extracellular and soluble proteins and with the cell wall.

The pharmacological mechanism underlying the antimicrobial actions of the two isolated compounds is unknown. Our results suggest that these compounds may act by damaging the membrane and/or cell wall function. Their activity may also be due to the presence of the phenyl group and/or the alcohol function.

## 4. Experimental

### 4.1. Plant Material

*R. raetam* flowers were collected in 2009 in Tunisia (Kerker). Identification was carried out by Chaieb Mohamed (Department of Botany, Faculty of Sciences University of Sfax, Sfax, Tunisia). The voucher specimen (deposit number RR009) was deposited in our laboratory (Monastir) for future reference.

### 4.2. Extraction and Isolation

The flowers were air-dried for several weeks. Powdered plant tissues (700 g) were extracted three times by maceration with 3 L methanol (27 °C, 3 days) and the resulting extract was concentrated under reduced pressure. The methanol extract was successively extracted with equal volumes of four organic solvents of increasing polarity: petroleum ether, chloroform, ether-ethyl acetate and butanol. The ethyl acetate extract (30 g), was subjected to normal phase column chromatography (7.5 × 100 cm column) over silica gel (400 g) and eluted with ether-ethyl acetate gradient elution (9:1 ĸ 8:2 ĸ 7:2 ĸ EtOAc) in increasing order of polarity. The elutes were combined based on the TLC results and 20 fractions (F1 to F20) were ultimately obtained. Fraction F10 was further again subjected to chromatography over silica gel and eluted with CH_2_Cl_2_-EtOAc (9.5-0.5) to give compound **2** (20 mg). Fraction F14 was purified over silica gel and eluted with acetone to give compound **1** (5 mg).

### 4.3. Spectral Analyses

The ^1^H-NMR spectra were recorded on a Bruker AMX-300 instrument, operated at 300 MHz, and TMS was used as a internal standard. The ^13^C-NMR spectra were recorded on a Bruker AMX-300 instrument operated at 75 MHz. The chemical shift and coupling constants (J) values are reported in ppm (δ) units and Hz, respectively.

### 4.4. Structure Identification

Compound **1** was identified as licoflavone C (Figure 1). It has the following physical characteristics: Yellow powder (MeOH); C_20_H_18_O_5_; HR ESI-MS *m/z*, 338.1154 ([M^+^]); ^1^H-NMR (CD_3_OD) δ (ppm): 13.16 (1H, s, H-5-OH), 7.65 (H-2, d, H-2', H-6'; *J*_2'–3'_ = *J*_6'–5'_ = 8.7 Hz), 6.75 (H-2, d, H-3', H-5'; *J*_3'–2'_ = *J*_5'–6'_ = 8.7 Hz), 6.37 (H-1, s, H-3), 6.08 (H-1, s, H-6), 5.07(H-1, m, H-2"), 3.33 (H-2, d, H-1"; *J*_1"–2"_ = 5.4 Hz), 1.67 (H-3, s, H-4"), 1.56 (H-3, s, H-5"); ^13^C-NMR (CD_3_OD) δ (ppm): 21.1 (CH_3_; C-4"), 25.5 (CH_3_; C-5"), 28.5 (C-1"), 102.4 (C-6), 106.3 (C-3), 108.1 (C-4a); 111.0 (C-8), 119.8 (C-3', C-5'), 126.4 (C-1'), 126.7 (C-2"), 132.3 (C-2', C-6'), 135.4 (C-3"), 159.3 (C-4'), 163.6 (C-8a), 165.5 (C-5), 166.2 (C-2), 168.9 (C-7), 187.0 (C-4).

Compound **2** was identified as derrone (Figure 2). It has the following physical characteristics: Brown powder (MeOH), C_20_ H_16_O_5_; ESI-MS *m/z*, 336.0998 ([M^+^]), ^1^H-NMR (CD_3_OD) δ (ppm): 13.21 (1H, s, H-5-OH), 7.50 (H-1,s, H-2), 6.93 (H-2, d, H-2', H-6'; *J*_2'–3'_ = *J*_6'–5'_ = 8.7 Hz), 6.48 (H-2,s, H-3', H-5'; *J*_3'–2'_ = *J*_5'–6'_ = 8.7 Hz), 6.25 (H-1, d, H-4"; *J*_4"–3"_ = 10.2 Hz), 5.89 (H-1, s, H-6), 5.25 (H-1,d , H-3"; *J*_3"–4"_ = 10.2 Hz), 1.06 (H-6, s, 2 × CH_3_). ^13^C-NMR (CD_3_OD) δ (ppm): 29.5 (2 × CH_3_), 79.4 (C-2"), 95.3 (C-6), 107.0 (C-8), 107.5 (C-4a), 116.8 (C-4"), 117.0 (C-3', C-5'), 123.5 (C-1'), 125.3 (C-3), 129.9 (C-3"), 131.8 (C-2', C-6'), 154.8 (C-2), 58.0 (C-4'), 159.06 (C-8a), 159.09 (C-5), 161.2 (C-7), 182.8 (C-4).

### 4.5. Microrganisms

The microorganisms tested in this study were *S. aureus*(ATCC 25923), *E. coli* (ATCC 25922), *P. aeruginosa* (ATCC 27950), and *E. faecalis* (ATCC 29212). The yeasts were *C. albicans* (ATCC 90028), *C. glabrata* (ATCC 90030), *C. parapsilosis* (ATCC 22019), and *C. kreusei* (ATCC 6258).

### 4.6. Determination of Antibacterial and Antifungal Activities

#### 4.6.1. Disc Diffusion Method

The disc diffusion method [16] was applied to cultures growing for 18 h at 37 °C and adjusted to approximately 10^6^ CFU/mL. Five hundred mL of the inoculi were spread over plates containing Muller-Hinton Agar, and a paper filter disc (6 mm) impregnated with 25 or 50 µg/disc of the product (compound **1** or **2**) was placed on the surface of the media. The plates were left for 30 min at room temperature to allow the diffusion of the compounds, and then they were incubated at 37 °C for 24 h. Then the inhibition zone around the disc was measured with a pair of callipers. Two controls were also included in the test. The first involved the presence of dimethyl sulfoxide and the second, standard antibiotics (gentamycin and amphotericin B) that were used in order to control the sensitivity of the tested microorganism. Each test was carried out in triplicate.

#### 4.6.2. Micro-Well Dilution Assay

Minimum inhibitory concentration (MIC) values were determined by a micro dilution method [7]. The inocula of the bacteria and yeasts were prepared from 12 h broth cultures and suspensions were adjusted to 0.5 McFarland standard turbidity. Media were placed into each 96 wells of the microplates. Test solutions at 500 µg/mL were added in the first rows of the microplates and two-fold dilution of the compounds (250-–0.125 µg/mL) were made by dispensing the solutions to the remaining wells. Ten microliter culture suspensions were inoculated into all the wells. Ethanol-hexane (1:1) using 1% Tween 80, pure microorganisms and pure media were used in control wells. The test was carried out in triplicate in each run of the experiments. The plate was covered with a sterile plate sealer and then incubated for 18 h at 37 °C. The MIC was defined as the lowest concentration of the compounds that inhibits the growth of microorganisms after incubation.

#### 4.7. Cytotoxic Activity

Cytotoxicity was evaluated with the MTT test. This test is based on the reduction of 3-[4,5-dimethylthiazol-2-yl]-2,5-diphenyl tetrazolium bromide (MTT) in living cells which can be measured colorimetrically. The assay [25] was performed in 96-well plates using Hep-2 cells that are derived from a laryngeal carcinoma cell line. Cells were seeded in 96-well plates at a concentration of 105 cells/well and incubated for 24 h at 37 °C in a 5% CO_2_ enriched atmosphere. After treatment with various concentrations of each compound, the cells were incubated for an additional 48 h at 37 °C. Then the medium was removed and cells in each well were incubated with 200 µL of MTT solution (5 mg/mL) for 2 h at 37 °C. The MTT solution was then discarded and 200 µL insoluble formazan crystal was added. The optical density (OD) was measured at 540 nm. Data were obtained from triplicate wells. The cytotoxicity index (CI%) was calculated according to the following equation: CI% = (1 − (T/C)) × 100, where T and C respectively represent the mean optical density of the treated group and vehicle control group. CI50 is defined as the concentration (µg/mL) of the substrate that causes 50% death of cells.

## 5. Conclusions

The two isolated flavonoids showed good antibacterial activity against *E. coli* and *P. aeruginosa* and important cytotoxicity against Hep-2 cells. They can possibly be used as antimicrobial agents in new drugs for the therapy of infectious diseases caused by pathogens, and eventually as anticancer drug. However, further research is necessary to establish the pharmacological mechanism of each compound.
